# Responses, Physiological and Molecular Mechanisms, and Mitigation Strategies of Grapevine Under Salt Stress

**DOI:** 10.3390/ijms27156692

**Published:** 2026-07-27

**Authors:** Ting Zheng, Hongying Li, Lingzhu Wei, Jiang Xiang, Jianhui Cheng

**Affiliations:** Institute of Horticulture, Zhejiang Academy of Agricultural Sciences, Hangzhou 310021, China; zhengting12309@163.com (T.Z.); hongying_li2002@yeah.net (H.L.); weilingzhu@zaas.ac.cn (L.W.); xiangjiang717@163.com (J.X.)

**Keywords:** grapevine, salt stress, ionic homeostasis, transcription factors, epigenetic regulation, non-coding RNA, salt-tolerant rootstock, viticultural mitigation technology

## Abstract

Soil salinization has become a major global abiotic threat restricting sustainable viticulture, especially in coastal and inland saline–alkali zones. Unlike cereal crops mainly suffering from sodium toxicity, grapevine (*Vitis vinifera* L.) is a typical chloride-sensitive woody perennial, subjected to superimposed damages of osmotic stress, ionic imbalance and secondary oxidative injury under saline conditions which severely suppress vegetative growth and degrade berry quality. This review systematically summarizes the multi-layered physiological adaptive mechanisms of grapevine against salt stress, including ion homeostasis maintained by salt overly sensitive (SOS), Na^+^/H^+^ exchanger (*NHX*) and chloride channel (*CLC*) transporter families, active accumulation of osmoprotectants, synergistic enzymatic and non-enzymatic antioxidant systems, and phytohormone crosstalk networks formed by endogenous phytohormones (abscisic acid, ABA; jasmonic acid, JA; salicylic acid, SA; brassinosteroid, BR) and small signaling molecules. We further elaborate comprehensive molecular regulatory cascades governing salt tolerance, covering core functional genes for ion transport, master transcription factor families WRKY, MYB, APETALA2/Ethylene Response Factor (AP2/ERF), NAC, basic helix–loop–helix (bHLH) and emerging epigenetic regulatory layers mediated by deoxyribonucleic acid (DNA) methylation, microRNAs (miRNAs), long non-coding RNAs (lncRNAs) and circular RNAs (circRNAs). In addition, we integrate four categories of field mitigation strategies for saline vineyards: germplasm improvement via salt-tolerant rootstock grafting, rhizosphere soil basal amendment, exogenous biostimulant regulation, and precision agronomic optimization. Current experimental systems do not fully recapitulate complex field combined-stress conditions, as most studies rely on laboratory single-salt stress simulation. Meanwhile, multi-omics, Clustered Regularly Interspaced Short Palindromic Repeats (CRISPR) gene editing and high-throughput phenotyping tools provide promising approaches to deepen our understanding of grape salt tolerance. This review constructs a comprehensive theoretical framework linking physiological responses, molecular regulatory networks and practical field technologies, offering systematic theoretical references and technical guidance for salt-tolerant germplasm innovation and environmentally sustainable viticulture on saline soils.

## 1. Introduction

Soil salinization, a major abiotic stress threatening global agricultural sustainability, currently affects 20% of irrigated arable lands. Due to rising sea levels, improper irrigation and extensive land use, salt-affected areas are continuously expanding, with over 50% of global cultivated lands projected to suffer saline–alkali stress by 2050 [1]. As an economically vital fruit crop worldwide, grapevine (*Vitis vinifera* L.) exhibits suppressed vegetative growth and reduced yield of high-quality berries under vineyard soil salinization. China possesses 1.5 billion mu of saline–alkali land distributed across coastal tidal flats, inland areas of the northwest, and protected farmlands; this situation is a representative regional example of this global challenge, with soil salinization severely restricting large-scale cultivation of specialty fruit trees [2]. Based on formation mechanisms and phytotoxic characteristics, salt stress is categorized into three types: neutral salt-induced stress that inhibits growth via osmotic and ionic toxicity, alkaline salt-triggered stress that damages roots and causes nutrient deficiency by elevating soil pH, and combined saline–alkali stress that exerts the most severe synergistic damage to plants [3]. This review focuses on NaCl-dominated neutral salt stress, in which Cl^−^ toxicity acts as a critical damaging factor for grapevines.

In the dominant protected grapevine cultivation systems, unreasonable fertilization and irrigation practices readily trigger secondary soil salinization. The high-temperature, humid, and highly evaporative microenvironment within greenhouse and rain shelter facilities degrades soil properties and exacerbates salt accumulation with long-term cultivation. Coastal vineyards also suffer from severe salt accumulation in dry seasons due to high groundwater tables; meanwhile, climate change-driven warming, reduced rainfall, and elevated evapotranspiration further exacerbate rhizosphere salt enrichment. Excessive accumulation of soluble salts (NaCl, Na_2_SO_4_, NaHCO_3_, and Na_2_CO_3_) leads to continuous soil saline–alkali degradation. Abundant Na^+^, Cl^−^ and other ions in salinized soils competitively inhibit the absorption of water, Ca^2+^ and K^+^ by grapevine roots, disturbing plant metabolism, weakening vine vigor and reducing yield [4]. Maintaining intracellular K^+^/Na^+^ homeostasis and eliminating excess cytoplasmic Na^+^ are two core salt-tolerance mechanisms in plants. Toxic ions are transported upward through the xylem transpiration stream, and the efficiency of apoplastic, symplastic and transmembrane transport pathways ultimately governs salt accumulation and salt damage severity in grapevine [4].

Grapevine possesses distinct salt-tolerance mechanisms compared with cereal crops. Unlike cereals that suffer mainly from Na^+^ toxicity, grapevine is a typical Cl^−^-sensitive woody perennial, with leaf Cl^−^ accumulation exceeding Na^+^ under salt stress. Chloride abundance is therefore a key index for assessing grape salt-tolerant germplasm [5]. Different from non-grafted cereal crops, commercial grapevines rely on a rootstock–scion system, in which substance exchange and long-distance signal crosstalk form a unique regulatory network governing salt tolerance [6]. Given the combined Na^+^/Cl^−^ dual toxicity and specific rootstock–scion interactions, conventional crop salt-tolerance theories cannot fully explain grapevine salt responses, highlighting the necessity of systematic and targeted research.

Previous studies have accumulated substantial physiological and molecular data on grapevine salt stress, whereas existing reviews remain fragmented. Most existing studies focus separately on physiological responses, rootstock screening or individual gene functions, lacking an integrated framework linking physiological phenotypes, coding genes, non-coding RNAs and field improvement strategies. Moreover, previous reviews generally overlook the unique biological traits of grapevine and fail to establish a systematic salt-tolerance regulatory network. To fill these research gaps, this review systematically elaborates salt-induced damage and endogenous defense mechanisms, integrates multi-level regulatory networks of ion transporters, transcription factors and non-coding RNAs, and summarizes field mitigation measures involving rootstock selection, soil amelioration, exogenous regulation and agronomic optimization. This review bridges theoretical and applied research, providing systematic theoretical and technical support for salt-tolerant germplasm breeding and sustainable green cultivation of grapevines in saline vineyards.

## 2. Multi-Tiered Damaging Effects of Salt Stress on Grapevine

Salt-induced injuries to grapevine exhibit distinct temporal, hierarchical and cumulative characteristics. Damage proceeds sequentially through early salt signal perception and signal transduction [7], followed by early osmotic stress, mid-term ionic toxicity and late secondary oxidative damage. These multi-layered injuries interact synergistically to form a vicious cycle, ultimately suppressing vine growth and degrading berry quality.

### 2.1. Osmotic Stress Injury (Early Primary Damage)

The lowered water potential of saline soils directly triggers osmotic stress in grapevine roots, impeding water uptake, restricting leaf gas exchange via stomatal closure, and inhibiting photosynthetic carbon assimilation at the initial stress stage [1]. As the initial salt response that occurs prior to ion accumulation, osmotic injury is mainly regulated by long-distance root-to-shoot abscisic acid (ABA) signaling. Salt stress drastically alters physiological and photosynthetic parameters in grapevines. Stomatal resistance, relative electrical conductivity, malondialdehyde (MDA) content, ferric reducing antioxidant power (FRAP) antioxidant capacity and ABA concentration are markedly elevated, alongside increased chlorophyll fluorescence indices (ABS/RC, TRo/RC, and DIo/RC). In contrast, leaf relative water content, photosynthetic pigment content, maximum photochemical efficiency (Fv/Fm), performance index (PI–abs) and electron transport efficiency (ETo/RC) continuously decline [8,9].

Photosynthetic suppression during this early phase is dominated by stomatal limitation: root osmotic imbalance reduces cell turgor and induces ABA-dependent stomatal closure, which lowers stomatal conductance and intercellular CO_2_ concentration to suppress photosynthetic efficiency. Diminished leaf photosynthesis curtails assimilate supply; since grape berries fully rely entirely on leaf-derived carbon resources, impaired leaf carbon fixation ultimately causes carbohydrate starvation and halts berry development [10].

### 2.2. Ionic Toxicity and Nutritional Imbalance (Mid-Term Primary Damage)

Following osmotic stress, excessive soil Na^+^ and Cl^−^ are transported to grapevine aerial tissues and induce severe ionic toxicity as well as nutritional disorders. Unlike most cereal crops, which suffer primary damage from Na^+^, grapevines are typical Cl^−^-sensitive fruit trees. Their leaves accumulate far higher concentrations of Cl^−^ than Na^+^, and chloride abundance directly correlates with salt injury phenotypes; this is a unique salt-responsive trait specific to grapevines [11]. Salt stress drastically elevates Na^+^ and Cl^−^ concentrations in grapevine roots and leaves, with these two ions showing an extremely significant positive correlation. Toxic monovalent Na^+^ competes for non-selective cation channels shared by multiple cations to block K^+^ influx; it further suppresses the uptake of divalent cations (Ca^2+^ and Mg^2+^) and trivalent trace elements (Fe, Zn, and Mn) by disrupting root membrane potential and inhibiting the activity of ion-specific channels and transporters responsible for these nutrients [12]. This disrupts intracellular K^+^/Na^+^ homeostasis, compromises membrane stability and enzymatic activity, and disturbs whole-plant nutrient metabolism [13,14,15]. Leaf Na^+^ and Cl^−^ levels continuously rise alongside increasing salinity and peak at 100 mM NaCl, which further aggravates salt damage [14].

As a glycophytic C3 plant, grapevine has no sodium requirement to sustain normal growth, in contrast to halophytic C3 species that rely on sodium to achieve optimal growth [16]. Excessive cytoplasmic and chloroplastic Na^+^ accumulation directly destroys photosynthetic structures and disrupts physiological metabolism, triggering non-stomatal photosynthetic inhibition via damaging PSII reaction centers and interrupting photosynthetic electron transport [11,17]. Grapevine exhibits moderate salt sensitivity without a unified tolerance threshold: growth inhibition initiates above 50 mM NaCl, while yield and berry quality degrade drastically beyond 100 mM NaCl. Tolerant rootstocks (‘101-14’, ‘1103 P’) can withstand 100~130 mM NaCl through effective root ion sequestration [18].

Grapevine roots possess strong Na^+^ retention capacity, and compartmentalized ion storage within root tissues serves as an essential foundation for salt tolerance. Salt-tolerant rootstocks efficiently restrict long-distance translocation of Na^+^ and Cl^−^, reduce ion accumulation in scions and berries, alleviate ionic antagonism, and maintain whole-plant nutritional homeostasis. This represents a vital strategy to mitigate salt stress in grape cultivation [19].

### 2.3. Oxidative Stress and Cellular Structural Damage (Secondary Injury)

Sustained osmotic and ionic stress disrupt cellular reactive oxygen species (ROS) homeostasis in grapevine. Excess ROS are predominantly generated from chloroplast photosynthetic electron transport chains and mitochondrial respiratory chains, resulting in massive ROS accumulation within chloroplasts and mitochondria and triggering severe oxidative damage [11]. While low basal ROS levels function as stress signaling molecules to activate defense responses, overproduced ROS under salt stress attack proteins, lipids and nucleic acids, induce membrane lipid peroxidation, destroy cellular and organelle structures, and promote the buildup of toxic metabolites such as MDA [2].

Grapevine scavenge surplus ROS through synergistic antioxidant systems, including enzymatic antioxidants superoxide dismutase (SOD), catalase (CAT) ascorbate peroxidase (APX) and guaiacol peroxidase (POD), as well as non-enzymatic (glutathione and flavonoids), to mitigate oxidative injury [1]. The antioxidant defense machinery can sustain cellular homeostasis under mild salt stress; however, prolonged or high-salinity stress depletes antioxidant pools, collapses the antioxidant defense system, and exacerbates oxidative damage. This stress injury further propagates into fruit tissues, disrupts the metabolism of cell wall pectin and cellulose, compromises cell wall integrity, and ultimately induces premature berry softening, which impairs fruit storability and commercial value [20].

### 2.4. Dual Suppression of Plant Growth and Berry Quality

Multi-layer salt injuries ultimately retard vine growth, reduce yield and deteriorate berry quality, affecting the entire vegetative and reproductive developmental cycle of grapevines. In terms of vegetative growth, salinity suppresses chlorophyll synthesis and disrupts chloroplast ultrastructure, weakening photosynthetic carbon fixation and organic matter accumulation [9]. Salt-stressed vines frequently exhibit stunted growth, scorched chlorotic leaves, and brown degenerated roots with severely impaired nutrient uptake, which drastically diminishes vine vigor [17,19]. Field trial statistics for own-rooted ‘Sultana’ grapevines demonstrated that each 1 mS/m elevation in soil electrical conductivity can induce up to 9% grape yield loss under the tested cultivation regimes [11].

Salt stress exerts bidirectional influences on fruit quality with an overall deteriorating tendency. Mild salinity (40 mM) slightly elevates berry concentrations of sugars, phenols, tannins and anthocyanins. In contrast, moderate and high salinity lowers fruit set, restricts pigment biosynthesis, and disturbs the sugar–acid balance. Residual Na^+^ and Cl^−^ accumulated in berries disrupt flavor profiles, generating diminished fruity aroma and oxidative off-flavors [2,21]. To mitigate oxidative damage, grapevines actively accumulate secondary metabolites including resveratrol and piceid under salt stress [22]. Physiologically, this mild salinity-induced flavor improvement arises from synchronized accumulation of osmoregulatory compounds and flavor-associated secondary metabolites. Moderate salt regulation therefore bears practical application potential for targeted quality improvement in commercial vineyard production.

## 3. Endogenous Physiological Regulatory Mechanisms of Salt Tolerance in Grapevine

Salt stress induces multiple types of damage in grapevines via water deficit, ionic toxicity, and intracellular ion imbalance. Grapevine activates multi-layered endogenous physiological defense systems to adapt to saline environments, relying on coordinated pathways including ionic homeostasis maintenance, osmoprotectant accumulation, antioxidant detoxification, and endogenous hormone signaling [23]. This section elaborates diverse endogenous physiological regulatory strategies adopted by grapevines under salt stress.

### 3.1. Ionic Homeostasis Regulation

Maintaining ionic homeostasis acts as the fundamental physiological mechanism underlying salt tolerance in grapevines. Plants employ two core strategies, salt exclusion and ion compartmentalization, to balance intracellular ion concentrations. This process primarily depends on the salt overly sensitive (SOS) pathway, which drives cytoplasmic Na^+^ efflux, and tonoplast-localized NHX-type Na^+^/H^+^ antiporters that mediate vacuolar ion sequestration [24].

#### 3.1.1. Ion Regulatory Characteristics of Grapevine and Rootstocks

Unlike cereal crops that mainly tolerate Na^+^ stress, salt resistance in grapevines relies on coordinated regulation of both Na^+^ and Cl^−^. Salt-tolerant rootstocks stabilize ionic balance through two approaches: restraining root salt uptake to reduce upward ion translocation, and compartmentalizing absorbed toxic ions to limit their accumulation in photosynthetic and reproductive functional organs. Rootstock genotypes exhibit drastic differences in ion regulation capacity. The salt-tolerant rootstock ‘110R’ displays significantly higher leaf *VviNHX1* transcript abundance than the salt-sensitive ‘1613 C’, and elevated expression of this gene positively correlates with the salt-resistant phenotype characterized by low leaf Na^+^ and a high K^+^/Na^+^ ratio [25]. Compared with salt-sensitive genotypes, tolerant grapevines sequester more Na^+^ and Cl^−^ within root tissues to alleviate ion buildup and salt injury in photosynthetic tissues [26].

Excessive cytoplasmic Na^+^ under salinity competitively blocks Ca^2+^ influx. It occupies non-selective cation channels (NSCCs) and depolarizes the root plasma membrane to suppress voltage-gated Ca^2+^ channels, while competing with K^+^ for shared uptake transporters; this dual inhibitory effect disrupts essential cation absorption and triggers ionic imbalance, leaf chlorosis, suppressed photosynthesis and growth arrest. Grapevine remodels metabolic allocation to slow redundant root growth and cut energy expenditure, diverting more resources toward salt-resistance pathways and reinforcing root Na^+^ retention [2]. A high cytoplasmic K^+^/Na^+^ ratio has been validated as a critical biomarker for sustained ionic homeostasis and enhanced salt tolerance [27].

#### 3.1.2. SOS Pathway-Mediated Na^+^ Efflux and Vacuolar Ion Compartmentalization

The SOS cascade represents the most thoroughly characterized core pathway governing plant salt stress responses [28]. Triggered by salt-induced cytoplasmic Ca^2+^ signals, this pathway sequentially activates Na^+^ efflux cascades.

Vacuolar ion sequestration acts as a vital intracellular detoxification strategy for grapevines. Confining surplus Na^+^ within vacuoles lowers cytoplasmic ionic toxicity, reduces cellular osmotic potential to strengthen water retention, and simultaneously alleviates both ionic and osmotic stress [29,30,31]. This process is governed by tonoplast Na^+^/H^+^ antiporters, which shuttle cytoplasmic Na^+^ into vacuoles for storage relying on the transmembrane proton gradient generated by vacuolar H^+^-ATPases and H^+^-pyrophosphatases [32,33,34].

These two pathways function synergistically: the SOS pathway restricts Na^+^ entry into root cells, whereas vacuolar compartmentalization sequesters absorbed Na^+^, jointly maintaining cytoplasmic Na^+^ homeostasis in grapevines.

Temporally, grapevine salt responses are divided into an early osmotic stress phase and a late ionic toxicity stage. Ionic homeostasis regulation persists through the entire stress process, coordinating with osmotic adjustment and antioxidant defense to construct a sequential multi-layered physiological network of salt tolerance [35].

### 3.2. Active Accumulation of Intracellular Osmoprotectants

To mitigate osmotic deficit damage during the early salt stress phase, grapevines actively synthesize and accumulate compatible solutes including soluble sugars, proline, and glycine betaine. By elevating intracellular solute concentration, these metabolites lower cellular osmotic potential to establish a water potential gradient that sustains cell turgor and curtails transpirational water loss [17]. Grape-specific polyphenols such as resveratrol exert dual functions in osmotic protection and antioxidation, forming a unique salt-adaptative strategy exclusive to grapevines [36].

Salt stress drastically induces massive accumulation of proline and soluble sugars in grapevine leaves, and their concentrations show a significant positive correlation with photosynthetic pigment levels. Appropriate exogenous nitrogen supply remodels endogenous signaling pathways, upregulates the expression of pivotal regulatory genes (*VvWRKY28* and *VvDOF8*), accelerates proline biosynthesis, and improves osmotic adjustment capacity as well as overall salt resistance [37,38,39].

### 3.3. Synergistic Detoxification of Enzymatic and Non-Enzymatic Antioxidant Systems

Ionic imbalance and osmotic deficit under salt stress trigger massive ROS overaccumulation, which causes membrane lipid peroxidation and organelle injury. Grapevine activates coordinated enzymatic and non-enzymatic antioxidant systems to scavenge surplus ROS and maintain intracellular redox homeostasis [40].

The enzymatic antioxidant system centers on SOD, POD, CAT and APX; among these enzymes, APX acts as a core component of the Mehler water-water cycle to decompose cytoplasmic and plastidic H_2_O_2_ and sustain cellular redox balance under saline conditions [41]. The non-enzymatic system mainly relies on the ascorbate (AsA)–glutathione (GSH) cycle and polyphenol compounds [42]. Sixteen genes associated with the AsA–GSH cycle have been identified within the grapevine genome. Salt stress markedly upregulates core functional genes including *VvAPX6/7/8*, *VvDHAR1*, *VvMDHAR2* and *VvGR2* to efficiently eliminate cellular ROS. Heterologous overexpression of these grapevine genes significantly enhances salt tolerance in recipient plants, confirming the central role of this detoxification pathway [43]. Furthermore, flavonoids, anthocyanins, and proline collectively participate in non-enzymatic antioxidation and further strengthen grapevine tolerance against oxidative damage [44].

### 3.4. Crosstalk Regulation of Endogenous Phytohormones and Signaling Molecules

Endogenous phytohormones and small-molecule signal molecules form a hierarchical, interconnected regulatory network rather than acting independently. They coordinately modulate three core defensive pathways, ionic homeostasis, osmotic adjustment and antioxidant defense, to precisely balance grapevine growth and salt stress resistance. This network centers on ABA, with jasmonic acid (JA), salicylic acid (SA), gibberellin (GA), and brassinosteroid (BR) acting as cooperative or antagonistic regulatory hubs, while melatonin, nitric oxide (NO) and γ-aminobutyric acid (GABA) serve as upstream signaling messengers [45,46].

ABA serves as the master hormone governing grapevine salt responses. Salt stress rapidly triggers endogenous ABA accumulation. Signals are transmitted through the conserved PYR/PYL/RCAR–PP2C–SnRK2 cascade, which activates downstream AREB/ABF transcription factors to induce stomatal closure and stress-related gene expression, thereby reducing transpiration water loss [47,48]. Salt-tolerant rootstocks accumulate markedly higher ABA concentrations than sensitive genotypes. Exogenous ABA boosts antioxidant enzyme activity and facilitates the biosynthesis of proline, soluble sugars and flavonoids [5]. Heterologous overexpression of the grape ABA receptor gene *VaPYL4* activates ABA-dependent antioxidant pathways to alleviate membrane lipid peroxidation and improves salt tolerance [49].

Other phytohormones constitute a complementary regulatory network that interacts with the ABA pathway to fine-tune salt responses. JA activates phenylpropanoid metabolism and *NHX1* expression in grapevine to enhance vacuolar Na^+^ compartmentalization, and *Vitis rupestris* exhibits more sensitive responses to JA signals [50]. The core ethylene transcription factor EIN3 directly activates SOS1 and SOS2, establishing a direct molecular link between hormonal signals and the salt overly sensitive ion transport pathway [51]. Salicylic acid (SA) and brassinosteroid (BR) synergistically regulate water absorption, chlorophyll synthesis, and antioxidant capacity, mediating the growth–stress resistance trade-off [52,53]. *VvGA2ox7* positively activates ion transport, osmotic adjustment and antioxidant pathways to mitigate salt-induced cell membrane damage, revealing the functional role of GA metabolism in salt adaptation [54]. As upstream signaling messengers, melatonin and NO promote ABA and JA biosynthesis and trigger the AsA–GSH antioxidant cycle to sustain intracellular K^+^/Na^+^ homeostasis. Rather than operating in isolation, these signal molecules interact at multiple transcriptional and physiological layers, integrating a full suite of salt-tolerant physiological pathways into a unified endogenous regulatory system for grapevines.

**Figure 1:**
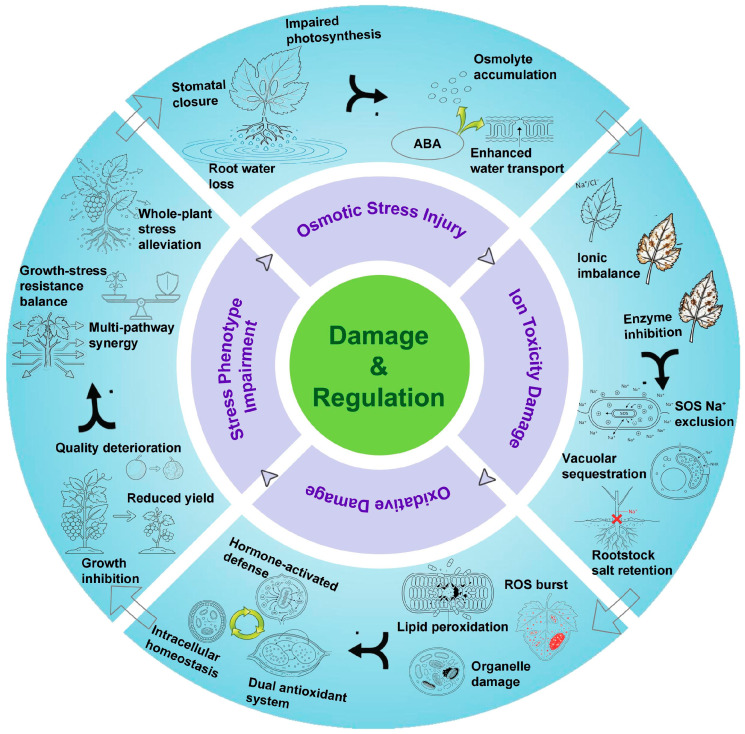
Model illustrating four categories of salt stress injury and corresponding grapevine-specific stress-alleviation regulatory mechanisms unique to *Vitis*. The circular schematic is composed of three concentric layers. The central panel outlines the core logic governing grapevine salt stress responses: damage onset and defensive regulation. The inner pale purple fan-shaped segments categorize four primary types of salt-induced injuries. The outer pale blue panels separately display physiological damage phenotypes triggered by each injury, alongside endogenous regulatory pathways through which grapevines resist stress and mitigate cellular harm. Collectively, this figure presents an integrated response framework of grapevines under salt stress that links damage initiation to downstream defensive regulation.

To systematically visualize the complete physiological cascade of damage occurrence and endogenous defense in grapevines under salinity, we constructed a schematic regulatory network, as summarized in Figure 1.

## 4. Molecular Regulatory Networks of Salt Tolerance in Grapevine

Under salt stress, grapevines coordinate signal transduction, gene expression, and metabolic reprogramming via multi-layered molecular regulatory networks to establish an integrated tolerance system that balances ionic homeostasis, ROS scavenging, osmotic adjustment, and vegetative growth. This regulatory system encompasses four core modules: ion transporters, transcription factors, non-coding RNAs and epigenetic modifications; these modules have emerged as dominant research hotspots in the molecular biology of grapevine abiotic stress over recent decades.

### 4.1. Key Functional Genes Regulating Ion Transport and Antioxidant Pathways

Transcriptomic and metabolomic analyses reveal that NaCl stress drastically remodels gene transcription and metabolite accumulation in grapevine roots. Numerous genes associated with flavonoid, phenylpropanoid, terpenoid and carbohydrate biosynthesis are strongly induced to coordinate antioxidant defense and osmotic adjustment [55]. Genes governing Na^+^ and Cl^−^ transport act as core functional components sustaining ionic homeostasis in grapevine. The SOS cascade genes (*SOS1*, *SOS2*, and *SOS3*) and vacuolar Na^+^/H^+^ antiporters (*NHX1* and *NHX2*) mediate sodium transport and compartmentalization, while CLC–G and CLC–C participate in vacuolar chloride sequestration. The calcium sensor *SOS3* and its homolog *SCaBP8* perceive intracellular Ca^2+^ signals, then bind and activate the serine/threonine kinase SOS2 [56,57]. Phosphorylated SOS2 further stimulates the plasma membrane Na^+^/H^+^ antiporter SOS1. Relying on the transmembrane proton gradient generated by plasma membrane H^+^-ATPases, SOS1 actively extrudes surplus cytoplasmic Na^+^ out of cells to fundamentally alleviate Na^+^ toxicity. NHX transporters are tonoplast-localized proteins belonging to the CPA1 superfamily and represent core functional genes conferring plant salt tolerance [58]. After Apse et al. [32] cloned the first plant *NHX1*, salt-tolerant functions of NHX family genes have been validated in model crops including *Arabidopsis thaliana* and rice [59,60]. Eight NHX homologs have been identified within the grapevine genome, distributed across six chromosomes. Except for *VvNHX5*, all paralogs localize to the tonoplast to modulate vacuolar salt compartmentalization. These genes participate in both plant development processes and salt response, and most are markedly upregulated under saline conditions [61,62]. Heterologous expression of these transporters in diverse crop species reduces toxic ion accumulation in leaf tissues and elevates biomass and yield under salinity. Grapevine *VvNHX1* is induced by NaCl and KNO3, whereas *VvNHX6* responds to ABA and is repressed by polyethylene glycol (PEG) [62]. Ectopic overexpression of *VvNHX1* in potatoes elevates soluble sugar, K^+^ and Mg^2+^ concentrations, activates antioxidant systems, and restrains Na^+^ buildup, directly verifying its salt-tolerant function in heterologous plant systems [58].

Intracellular K^+^/Na^+^ balance largely depends on diverse potassium transporters. Salt stress markedly upregulates root *VvK1.1* and *VvHKT1;1* in grapevine [11]. Non-invasive micro-test technology (NMT) assays confirm that overexpression of *VviHKT1;7* facilitates Na^+^ efflux and K^+^ retention in roots to optimize ionic equilibrium [63]. The root-specific high-affinity potassium transporter *VvHAK5* responds to both potassium deficiency and salt stress. Tobacco transformation experiments demonstrate that *VvHAK5* enhances K^+^ absorption, stabilizes K^+^/Na^+^ homeostasis and boosts ROS scavenging capacity in heterologous plant systems. The VvSAP8–VvHAK5 regulatory module confers dual tolerance to low potassium and salt stress [64].

HKT1;1 acts as a master regulatory protein restricting upward translocation of Na^+^ and Cl^−^. Root salt excretion and vacuolar compartmentalization are coordinately controlled by multiple transporters: SOS1 and NPF proteins mediate root salt efflux; cation–chloride cotransporters (CCCs) mediate intracellular ion chelation; tonoplast Na^+^/H^+^, K^+^/H^+^ (NHX-type) and Cl^−^/H^+^ (CLC-type) exchangers sequester sodium and chloride inside vacuoles; and Shaker-type K^+^ channels *VvK1.1* and *VvK5.1* likely facilitate K^+^ loading into the root xylem to maintain a favorable K^+^/Na^+^ ratio [11]. Grapevine serves as an ideal model to dissect chloride transport mechanisms, with chloride exclusion governed by polygenic regulatory networks [65]. Combined multi-species grape genome-wide association study (GWAS) and quantitative trait locus (QTL) mapping identified the leaf chloride exclusion locus qClEx8.1 on chromosome 8, which encodes cation/H^+^ exchangers (CHXs) that eliminate excess Cl^−^ via phloem recirculation [11].

Multiple genetic pathways enhance salt tolerance by regulating ROS homeostasis. Salt-induced overexpression of *VvMAPK9* in grapevine calli and *Arabidopsis* improves growth performance under salinity by activating antioxidant enzymes to accelerate ROS clearance [66]. The arginine/serine-rich gene *VvSR34a* positively regulates salt tolerance by raising chlorophyll content and root activity while lowering MDA and electrolyte leakage; in contrast, *VvCOP9* exerts negative regulation through targeting *VvSR34a* for degradation [67]. Heterologous expression of trehalose synthase *VVTPPA* and annexin *VvANN8* suppresses excessive ROS accumulation and alleviates salt damage. Plant salt responses depend on coordinated crosstalk among multiple molecular cascades, mainly including ROS-dependent MAPK signaling, NAC transcription factor pathways, Ca^2+^-dependent SOS ionic transport, ERAD degradation, and ABA and BR hormone signaling [3,68,69]. Salt stress suppresses enzymatic activity, triggers ROS bursts, inhibits cell elongation and disrupts membrane and osmotic balance. These interconnected regulatory pathways synergistically counteract multifaceted injuries and relieve salt-induced growth retardation [70].

### 4.2. Core Regulatory Pathways of Stress-Responsive Transcription Factors

Transcription factors (TFs) act as central regulatory hubs for salt signaling in grapevines. They precisely govern the spatiotemporal expression of salt-tolerance genes by specifically binding to cis-elements within target gene promoters and assemble sophisticated stress regulatory networks via protein–protein interactions and downstream signaling cascades [71]. Grapevine salt-responsive TFs span diverse functional families: WRKY, MYB and AP2/ERF form the core groups, while NAC, LBD, CBF, bHLH, SBP and BES1 serve as critical complementary regulators. These TFs harbor both evolutionarily conserved functions across plant taxa and grape-specific regulatory mechanisms, and are involved in the entire salt response cascade via positive or negative transcriptional modulation.

Members of the WRKY family act as key positive regulators of grape salt tolerance. Salt stress strongly induces the transcript levels of *VhWRKY44*, *VvWRKY28*, *VvWRKY30*, *VlWRKY3* and *VvWRKY2* [37,72,73,74,75]. Among these, *VvWRKY28* activates ABA biosynthesis, ionic homeostasis and antioxidant pathways, elevates the contents of osmoprotectants and antioxidant enzymes, and alleviates membrane lipid peroxidation [37]. *VvWRKY30* facilitates proline and soluble sugar synthesis and enhances ROS scavenging capacity [73]. Transgenic lines overexpressing *VhWRKY44*, *VlWRKY3* or *VvWRKY2* display enhanced root growth, more robust osmotic adjustment, and elevated salt resistance. A small subset of WRKY members exert negative regulatory effects: *VvWRKY13* shows high transcript abundance in salt-sensitive cultivars. It suppresses vegetative growth under saline conditions via a distinct ABA subpathway independent of the canonical SnRK2–RD22 signaling cascade [76].

The MYB family bridges hormone signaling, ion transport, and metabolic pathways and predominantly exerts positive regulatory functions. Melatonin-induced MYB108A binds to the promoter of the ethylene biosynthetic gene *ACS1* to activate ethylene signaling and boost grape salt tolerance [77]. Multiple R2R3–MYB members exert complementary functions: *VvMYB6*, *VhMYB2* and *VvRAX82* reduce ROS and MDA accumulation and stabilize photosynthesis by modulating antioxidant systems, salt signaling and carbon metabolism, respectively [49,71,78]. *VvMYB103* is a positive salt-tolerance gene with significantly higher salt-induced expression in tolerant cultivars than sensitive ones. Heterologous overexpression of this gene enhances seed germination and plant vigor by sustaining water homeostasis, boosting antioxidant capacity, and alleviating salt-induced membrane damage [79].

Members of the AP2/ERF family display obvious functional divergence and rely on protein–protein interactions for regulation. VvERF1B interacts with VvMYC2 to synergistically activate the plasma membrane H^+^-ATPase gene *VvPMA10* and maintain ionic homeostasis under saline–alkali stress [80]. *VviERF105* positively modulates salt tolerance, and its protein stability is governed by the *VviPUB19*-mediated 26S proteasome pathway, whereas *VvARF6* negatively regulates salt resistance [81,82]. *VvERF2* promotes callus proliferation and phenolic compound accumulation to improve salt tolerance [83]. Although *VviERF073* has been confirmed as salt-inducible, its full function in salt stress resistance in grapevines remains poorly uncharacterized [84].

The LBD, NAC, CBF, bHLH, SBP and BES1 families constitute a complementary regulatory network with coordinated positive and negative regulatory effects. The LBD family is predominantly repressive: *VvLBD39* exacerbates salt damage in grapevines by inhibiting ABA biosynthesis, reducing ROS scavenging efficiency, and downregulating stress-responsive genes. Transgenic lines overexpressing this gene exhibit impaired photosynthetic capacity, delayed root development, diminished antioxidant capacity, and excessive ROS and MDA accumulation [85]. The NAC family mediates crosstalk between ABA and JA pathways; both *VvNAC17* and *VvMYC2* significantly improve salt tolerance in transgenic plant lines [86,87]. CBF/DREB, bHLH, SBP and BES1 families target antioxidant metabolism, flavonoid biosynthesis, SOS signaling cascades, and ABA signaling, respectively. Overexpression of *VaCBF4*, *VvbHLH1*, *VpSBP16* and *VvBES1–3* increases proline content and antioxidant enzyme activity, reduces MDA accumulation, and enhances salt tolerance via regulatory mechanisms [88,89,90,91,92].

Collectively, diverse transcription factors integrate ion transport, antioxidant defense, and hormone metabolism pathways via specific promoter binding, protein–protein interactions and signaling cascade activation, forming a unified molecular regulatory network that governs salt tolerance in grapevines.

### 4.3. Emerging Regulatory Roles of Epigenetic Modifications

Epigenetic modifications represent emerging core regulatory mechanisms governing plant abiotic stress responses, including DNA methylation, histone modification, histone variants and non-coding RNA regulation. They precisely shape the expression profiles of salt-responsive genes without altering the underlying genomic sequences [93]. To date, epigenetic studies on grapevine salt stress remain in the initial exploratory stage, and the associated regulatory pathways have not yet been systematically elucidated.

DNA methylation serves a critical epigenetic regulatory pathway for grapevine salt stress responses. Wang et al. [94] identified the methyltransferase gene *VvMET2b* in grapevines, marking the first functionally validated grape gene that confers salt tolerance by mediating DNA methylation. Salt stress upregulates *VvMET2b* and elevates global DNA methylation levels in grapevine tissues. Heterologous overexpression of *VvMET2b* significantly increases the seed germination rate and seedling survival under salt stress in the model plant *Arabidopsis thaliana*. Integrated methylome and transcriptome analyses demonstrate that *VvMET2b* reshapes the methylation patterns of key salt-tolerance genes (*MYB6*, *EXPA17*, and *TIP2*) to modulate their transcription and facilitate grapevine salt adaptation.

Non-coding RNAs form a multi-layered post-transcriptional regulatory network underlying grapevine salt tolerance. Through endogenous competitive sequestration, splicing suppression, and translational repression, they coordinate ionic homeostasis and hormone metabolism to collectively determine salt-tolerance phenotypes. miRNAs and lncRNAs represent two core classes of non-coding regulators. Wei et al. [95] identified 39 salt-induced differentially expressed miRNAs via high-throughput sequencing, including canonical salt-responsive miR390 and miR394. Jin et al. [96] detected 1661 salt-responsive lncRNAs in grapevines, confirming their widespread involvement in salt signal transduction.

Circular RNAs (circRNAs) are newly identified central regulators of grapevine salt stress with diverse and flexible regulatory modes. Gao et al. [97] demonstrated that it has been proven that plant circRNAs are generated via canonical spliceosomal pathways. Acting as an endogenous RNA (ceRNA), Vv–circSIZ1 sponges miR3631 and derepresses the downstream target gene *VvVHAc1*, positively regulating grape salt tolerance. Gao et al. [98] further characterized the stress-related functions of natural antisense circRNAs: VvcircABH inhibits pre-mRNA splicing of its host gene *VvABH*, modulates brassinosteroid signaling and alters grape salt susceptibility. The same research group identified the VvcircHMA1–VvmiR167b–VvARF6 regulatory cascade, in which the circRNA sequesters miR167b to derepress *VvARF6* and amplify stress signals via a positive feedback loop. Moreover, the novel circRNA Vv–circRCD1 drastically enhances salt resistance. Its heterologous expression remodels root architecture in *Arabidopsis*, and transient overexpression in grapevine also strengthens salt tolerance [99].

In summary, epigenetic regulation mediated by DNA methylation and non-coding RNAs finely tunes the expression of salt-tolerance genes at both the chromatin and post-transcriptional levels, constituting a newly identified, critical supplementary layer of the grapevine salt-tolerance regulatory network.

## 5. Field Mitigation Technologies for Grapevine Salt Stress

Soil salinization severely restricts large-scale cultivation and high-quality production of grapevines in saline–alkali regions. Developing eco-friendly and sustainable field techniques, including germplasm improvement, soil regulation, exogenous application and optimized cultivation practices, to alleviate salt damage represents the core strategy to boost yield and fruit quality of grape industries on saline lands.

### 5.1. Germplasm Genetic Improvement

Grapevine is classified as a moderately salt-tolerant fruit crop. Grafting onto salt-tolerant rootstocks serve as the primary germplasm improvement strategy to enhance salt resistance for sustainable grape production in saline–alkali areas [19,100]. Commercial rootstocks exhibit distinct gradations of salt tolerance, categorized into high-, moderate- and low-tolerance groups: ‘Salt Creek’, ‘Dogridge’, ‘140R’, and ‘1103P’ are highly salt-tolerant; ‘110R’ and ‘Rupestris du Lot’ show moderate tolerance; ‘5C’ and ‘Riparia Gloire’ are salt-sensitive [18,101,102]. Common rootstocks widely used in Chinese vineyards include ‘SO4’, ‘Beta’, ‘3309C’, ‘5BB’, and ‘1103P’. Among these, ‘SO4’ and ‘1103P’ are classified as high salt-tolerant genotypes, whereas ‘5BB’ and ‘3309C’ are susceptible to salinity [100,103].

Rootstock–scion crosstalk constitutes the central mechanism by which rootstocks modulate whole-plant salt tolerance. Rootstocks mediate long-distance transport of salt-tolerance signals and functional metabolites via vascular tissues. Mobile signals, including phytohormones, small RNAs, peptides and secondary metabolites, can travel across graft unions to coordinate systemic stress responses; this explains why scion salt phenotypes are strongly dependent on rootstock genotypes and enables the systemic transmission of salt resistance throughout the entire vine [6,104,105]. Grafting ‘Flame Seedless’ grape onto the highly tolerant ‘1103P’ rootstock significantly enhances overall salt resistance: grafted vines accumulate more K^+^ and less toxic Na^+^ and Cl^−^ to maintain ionic balance and relieve salt injury [102]. Recent studies further confirm that root-derived signal molecules can reprogram the transcriptional network of scion leaves, modulating ion transport and antioxidant capacity under saline conditions [106,107]; however, the identity of key long-distance signaling molecules in grape rootstock–scion systems remains largely unclear.

Comprehensive physiological analyses demonstrate that salt-tolerant rootstocks share three core resistant traits: root chloride exclusion, sodium sequestration and stable K^+^/Na^+^ equilibrium. Rootstocks restrict long-distance chloride translocation into the xylem to prevent photosynthetic impairment caused by chloroplast Cl^−^ accumulation. Meanwhile, tonoplast transporters compartmentalize excess root Na^+^ to sustain favorable shoot K^+^/Na^+^ ratios. Combined with long-distance signal communication between the rootstock and scion, these mechanisms collectively substantially improve salt tolerance at the whole-plant level.

Wild grape germplasm resources harbor abundant salt-tolerant genetic variations and serve as elite gene pools for rootstock breeding and genetic improvement. Muscadine grapes exhibit broader salt adaptability and develop milder chloride-induced leaf injury, with the cultivars ‘Janebell’ and ‘Scuppernong’ showing the strongest chloride exclusion capacity [108]. North American wild species, including *Vitis riparia*, *V. champinii* and *V. berlandieri,* represent core parental materials for breeding salt-resistant rootstocks. Tunisian wild grape (*Vitis sylvestris*) employs a unique salt-tolerance mechanism that relies on efficient redox homeostasis rather than root sodium sequestration [87]. Chinese wild *Vitis amurensis* outperforms European wine grapes and commercial rootstocks in terms of salt resistance. The representative cultivar ‘Shuangyou’ sequesters excess Na^+^ in root tissues, resulting in a 3.8-fold higher shoot K^+^/Na^+^ ratio than ‘Cabernet Sauvignon’ and demonstrating superior ionic regulatory ability [109]. Population genomic analysis of wild *Vitis heyneana* reveals that long-term adaptation to coastal high-salinity habitats drives positive natural selection of *FSD2*, *RGA1* and *AAP8*, which are core functional genes underlying salt-adaptive evolution [110]; these evolution-associated genes differ from the acute salt-response genes of cultivated grapes described in Section 4.

### 5.2. Rhizosphere Soil Improvement Through Basal Amendments

The rhizosphere refers to the microscale soil zone modulated by root exudates, and rhizosphere microbiota act as crucial microecological factors that mitigate salt-induced damage to grapevines. Rhizosphere microbial responses differ substantially across rootstocks with divergent salt-tolerance levels. The highly salt-tolerant rootstock ‘101-14’ enriches beneficial rhizospheric microorganisms to a far greater degree than the salt-sensitive rootstock ‘5BB’ and alleviates root ionic toxicity by strengthening plant–microbe interactions [100].

Biochar, humic acid, gypsum and organic mulches are widely adopted rhizosphere amendments in saline–alkali vineyards. As porous carbon-rich biomass with a high cation exchange capacity, biochar effectively adsorbs free soil Na^+^, facilitates root K^+^ retention and Na^+^ exclusion, and upregulates aquaporin gene expression to alleviate salt-triggered osmotic injury [111]. Humic acid improves both soil structure and plant stress-related metabolism, enhancing grape salt tolerance through combined edaphic and physiological regulation [112]. Gypsum application to sodic saline–alkali soils displaces colloidal-adsorbed Na^+^ and accelerates salt leaching downward salt leaching. Field straw and compost mulching limits salt accumulation in topsoil, preserves rhizosphere moisture, and stabilizes the root-zone microenvironment [11].

Rational combined fertilization and integrated bacteria–biochar amendments confer long-term rhizosphere salt resistance. Moderate nitrogen supplementation during the flowering period reduces flower and fruit abscission, increases cluster yield, and enhances grape salt tolerance [113]. Salt-tolerant bacterial strains, including *Bacillus subtilis* and *Pseudomonas resinovorans*, optimize the rhizosphere microenvironment via exopolysaccharide secretion. Co-application of functional microorganisms and biochar produces superior improvements in soil physicochemical properties and microbial community structure relative to single-component amendments [20].

### 5.3. Exogenous Chemical Regulation

Exogenous application of biostimulants and small-molecule signaling substances directly modulates endogenous metabolic pathways and rapidly alleviates salt-induced damage in grapevines, representing an efficient salt-tolerance regulation strategy separate and distinct from germplasm improvement and soil remediation [114]. Studies on berry crops have confirmed that a range of biostimulants including 5-aminolevulinic acid (5-ALA), melatonin (MLT), SA, JA, GABA, BR, ABA and trehalose, alongside nanoscale regulators such as zinc oxide nanoparticles, silicon nanoparticles, and proline-functionalized graphene oxide nanoparticles (GO-Pro NPs), can effectively alleviate salt-induced growth retardation in grapevines [14,44,115]. With respect to nanomaterial applications, foliar spraying of GO-Pro NPs under abiotic stress alleviates salt-induced oxidative stress in grapevines [44]. Among conventional biostimulants, trehalose mitigates salt-mediated oxidative injury [116]; 5-ALA alleviates leaf membrane damage, elevates ATPase activity, and enhances salt resistance of grape rootstocks [117]; and melatonin and the ethylene precursor 1-aminocyclopropane-1-carboxylic acid (ACC) significantly reduce oxidative damage to cell membranes under salt stress [77,118]. As a critical bioactive compound, dopamine exerts broad and potent regulatory effects against salt stress. Treatment with 0.4 mM dopamine substantially alleviates growth inhibition under high salinity by reducing ROS and MDA accumulation, mitigating membrane injury, maintaining ionic homeostasis, and concurrently improving photosynthetic efficiency and antioxidant enzyme activity, thereby conferring enhanced salt tolerance in grapevines via multi-pathway regulation [119].

Targeted studies on grapevine demonstrate that foliar application of exogenous GABA, SA and BR exerts unique bidirectional regulatory advantages. These regulators not only recover salt-inhibited vegetative growth, including impaired root activity and premature leaf senescence, but also rescue disrupted berry development and preserve intrinsic fruit quality. They effectively balance vegetative and reproductive performance under saline conditions, positioning them as promising exogenous regulators for high-quality grape production in saline–alkali regions [120].

### 5.4. Supporting Agronomic Practices

Precision agronomic management represents a low-cost, eco-friendly and sustainable strategy to mitigate salt damage without additional exogenous amendments. By optimizing field water and fertilizer regimes and the root-zone microenvironment, salt stress injury to grapevines can be fundamentally alleviated [5]. Irrigation management serves as the core measure for salt leaching. Prolonged single irrigation events facilitate vertical water leaching to wash accumulated Na^+^ and Cl^−^ out of the dense root zone and reduce rhizospheric salt buildup. Combined with field drainage ditches, rainfall can flush topsoil salts down to subsoil deeper than 1.5 m, beyond the active root absorption zone, and maintain a long-term low-salt rhizosphere environment [5]. The water-saving Partial Rootzone Drying (PRD) irrigation system tailored for saline vineyards remodels plant water signaling and redistributes toxic ions via alternate drying–wetting cycles of separated root compartments, simultaneously saving water, improving yield, and enhancing salt tolerance.

Root confinement cultivation and surface mulching serve as vital auxiliary salt-suppression approaches. Root confinement effectively inhibits salt backflow and reduces water–fertilizer leaching to achieve precise and efficient nutrient utilization. Inter-row grass cover, straw or plastic film mulching around tree trunks cuts soil water evaporation, blocks upward salt transport via capillary water, stabilizes the rhizosphere microenvironment and alleviates salt stress in grapevines.

**Figure 2:**
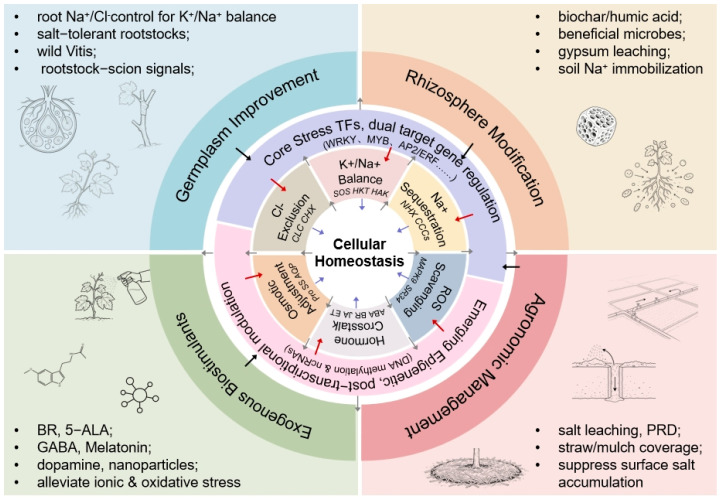
Schematic of multi-layered molecular regulatory network of grapevine under salt stress. The figure is divided into four colored sectors, representing four categories of field salinity mitigation strategies: germplasm improvement (blue), rhizosphere modification (tan), agronomic management (red), and exogenous biostimulants (green). Layers from outer to inner: field salinity mitigation techniques, transcription factor and epigenetic regulatory modules, six intracellular functional pathways, and central cellular homeostasis. Black arrows show field treatments regulating upstream molecular modules; red arrows indicate transcriptional and epigenetic modulation of downstream ion transport and antioxidant pathways; light purple converging arrows demonstrate synergistic maintenance of cellular homeostasis by six core molecular pathways; thin light-gray reverse arrows illustrate retrograde feedback from cellular homeostasis to regulate gene expression, molecular signals, and field-grown plant phenotypes, forming a closed regulatory circuit.

By integrating the intracellular molecular regulatory pathways and field salinity mitigation strategies discussed above, we constructed a multi-layer closed-loop regulatory network for salt-stressed grapevines, as summarized in Figure 2.

## 6. Current Research Bottlenecks and Future Perspectives

### 6.1. Existing Research Limitations

Current research on grapevine salt stress still has several key limitations. In terms of experimental design, most existing studies rely on laboratory-based single-salt stress simulation, an approach that fails to replicate the field conditions characterized by the combined stresses of salinity, drought and high temperature. Specifically, vineyards in field settings are exposed to drought, high temperature, and other co-occurring environmental factors, which are generally not incorporated into single-factor laboratory simulation assays. Moreover, previous research predominantly focuses on leaf physiological responses, while largely overlooking the temporal response dynamics of root reaction and fruit development under salt stress. At the molecular level, the in vivo salt-tolerant functions of non-coding RNAs remain poorly validated, and the long-distance regulatory mechanism of rootstock–scion interactions governing salt tolerance remains largely unclear. In terms of technical application, cutting-edge technologies including single-cell sequencing, in situ ion imaging, and high-throughput phenomics are insufficiently deployed, and the integrated multi-omics analytical framework remains underdeveloped. In addition, the evaluation of salt-tolerant germplasms is mostly limited to laboratory seedling experiments, which cannot accurately reflect field salt-resistance performance and thus fail to effectively support salt-tolerant grapevine breeding programs.

### 6.2. Future Research Prospects

To address the above limitations, future research will be comprehensively advanced across four targeted dimensions. Targeting the gap between laboratory single-factor simulation and field conditions, in-depth research on field combined-stress responses will be conducted, and precise water and fertilizer management technologies such as mulched drip irrigation and root-restricted cultivation will be widely deployed. When combined with soil amendments and organic fertilizer application for saline land ecological improvement, as well as rhizosphere microbial symbiosis regulation, a low-carbon and simplified cultivation model for saline vineyards will be established. Targeting incompletely characterized physiological and molecular regulatory mechanisms, integrated spatiotemporal multi-omics analysis and single-cell sequencing technology will be employed to elucidate the circRNA-mediated post-transcriptional regulatory network and the long-distance signaling interaction mechanism of rootstock–scion systems, and the research scope will be expanded from leaf responses to root reaction and fruit developmental characteristics to refine the theoretical framework of grapevine salt tolerance [5]. Targeting the limited application of cutting-edge detection technologies, high-throughput phenotyping, PET ion tracing and LA–ICP–MS in situ imaging will be integrated, and a standardized integrated multi-omics analytical system will be constructed to build a precise evaluation system for grapevine salt tolerance. Targeting the weak breeding support of germplasm evaluation, CRISPR gene editing, molecular marker-assisted selection and conventional breeding technologies will be integrated to overcome the bottleneck of low-efficiency genetic transformation and directionally breed high-quality, salt-tolerant grapevine rootstocks and cultivars, and digital field-based screening of salt-tolerant germplasms will be realized via smart agriculture technologies to provide reliable support for practical breeding programs [121].

### 6.3. Industrial Application Reflections

A prominent disconnection between theoretical research and industrial application remains prevalent in current grapevine salt-tolerance studies. Results obtained from laboratory simulation experiments are poorly adaptable to complex field saline environments, yielding limited guiding value for practical production. Furthermore, the grape industry faces a core trade-off between salt tolerance and fruit quality. Most salt-tolerant germplasms exhibit degraded fruit quality and diminished aroma traits, making it difficult to balance stress resistance and commercial value. Future research should be closely oriented toward production practices and correct the one-sided research bias of prioritizing salt tolerance over fruit quality. Elucidating the synergistic regulatory mechanisms between salt tolerance and fruit quality, and integrating molecular breeding, precision agronomy, and intelligent evaluation technologies, will facilitate the establishment of a high-quality, low-carbon cultivation system for grapevines in saline lands, providing robust support for the sustainable, high-quality development of the grape industry in saline–alkali regions.

## Data Availability

No new data were created or analyzed in this study. Data sharing is not applicable to this article.

## Abbreviations

The following abbreviations are used in this manuscript:

5-ALA	5-Aminolevulinic acid
ABA	Abscisic acid
ACC	1-Amino-1-cyclopropanecarboxylic acid
AP2/ERF	APETALA2/ethylene response factor
APX	Ascorbate peroxidase
bHLH	Basic helix–loop–helix
BR	Brassinosteroid
CAT	Catalase
CCCs	Cation–chloride cotransporters
CHX	Cation/H^+^ exchangers
ceRNA	Competing endogenous RNA
circRNA	Circular RNA
CLC	Chloride channel
CRISPR	Clustered regularly interspaced short palindromic repeats
DNA	Deoxyribonucleic acid
FRAP	Ferric reducing antioxidant power
GA	Gibberellin
GABA	γ-Aminobutyric acid
GO-Pro NPs	Proline-functionalized graphene oxide nanoparticles
GWAS	Genome-wide association study
JA	Jasmonic acid
lncRNA	Long non-coding RNA
MDA	Malondialdehyde
miRNA	MicroRNA
MLT	Melatonin
NHX	Na^+^/H^+^ exchanger
NMT	Non-invasive micro-test technology
NO	Nitric oxide
NSCCs	Non-selective cation channels
POD	Guaiacol peroxidase
PRD	Partial rootzone drying
QTL	Quantitative trait locus
ROS	Reactive oxygen species
SA	Salicylic acid
SOD	Superoxide dismutase
SOS	Salt overly sensitive
TFs	Transcription factors
